# Aspergillusidone G Exerts Anti-Neuroinflammatory Effects via Inhibiting MMP9 Through Integrated Bioinformatics and Experimental Analysis: Implications for Parkinson’s Disease Intervention

**DOI:** 10.3390/md23050181

**Published:** 2025-04-23

**Authors:** Fangfang Ban, Longjian Zhou, Zhiyou Yang, Yayue Liu, Yi Zhang

**Affiliations:** 1 Guangdong Provincial Key Laboratory of Aquatic Product Processing and Safety, Guangdong Provincial Engineering Laboratory for Marine Biological Products, Guangdong Provincial Center for Modern Agricultural Scientific Innovation, Shenzhen Institute of Guangdong Ocean University, Zhanjiang Municipal Key Laboratory of Marine Drugs and Nutrition for Brain Health, Research Institute for Marine Drugs and Nutrition, College of Food Science and Technology, Guangdong Ocean University, Zhanjiang 524088, China; banfangfang@126.com (F.B.); zyyang@gdou.edu.cn (Z.Y.); yayue_liu@163.com (Y.L.); 2 Southern Marine Science and Engineering Guangdong Laboratory (Zhanjiang), Zhanjiang 524088, China; 3 Collaborative Innovation Center of Seafood Deep Processing, Dalian Polytechnic University, Dalian 116034, China

**Keywords:** Parkinson’s disease, neuroinflammation, Aspergillusidone G, matrix metalloproteinase 9

## Abstract

Natural products have extensive attractiveness as therapeutic agents due to their low toxicity and high efficiency. Our previous study has identified a depside-type Aspergillusidone G (Asp G) derived from *Aspergillus unguis* DLEP2008001, which shows excellent neuroprotective activity for 1-methyl-4-phenylpyridinium (MPP^+^)-induced primary cortical neurons and anti-neuroinflammatory property, promising to be a potential therapeutic agent for Parkinson’s disease (PD). To further explore the anti-PD potential and mechanisms of Asp G, we employed network pharmacology, cellular experiments, and various biological techniques for analysis and validation. The analysis of network pharmacology suggested that Asp G’s anti-PD potential might be attributed to its modulation of inflammation. The data from nitric oxide (NO) detection, qRT-PCR, and Western blot confirmed that Asp G dose-dependently inhibited lipopolysaccharide (LPS)-stimulated NO production, with 40 μM Asp G suppressing 90.54% of the NO burst compared to the LPS group, and suppressed the overproduction of inflammatory-related factors in LPS-induced BV2 cells. Further protein–protein interaction analysis indicated that matrix metalloproteinase 9 (MMP9), a promising target for PD intervention, was the most likely anti-PD target of Asp G, and the results of gelatin zymography, qRT-PCR, and Western blot validated that Asp G could inhibit the active and inactive forms of MMP9 directly and indirectly, respectively. Notably, the inhibition of 67 kDa-MMP9 by Asp G is expected to compensate for the inability of TIMP-1 to inhibit this form. Furthermore, a selective inhibitor of MMP9 (20 μM SB-3CT) further potentiated the anti-inflammatory effects of Asp G (20 μM), with inhibition rate on NO increasing from 27.57% to 63.50% compared to LPS group. In summary, our study revealed that Asp G exerts anti-neuroinflammatory effects by inhibiting MMP9, which provides a valuable lead compound for the development of anti-neuroinflammatory drugs and offers insights into the intervention of PD-associated neuroinflammation. Future studies will further investigate the upstream regulatory mechanisms of Asp G-mediated MMP9 inhibition and its effects in in vivo PD models.

## 1. Introduction

Natural products attract considerable attention for their therapeutic potential in various diseases due to their structural diversity, multi-target effects, and generally favorable safety profiles [1,2,3]. Depsides, a group of naturally occurring phenolic compounds formed by the ester-bond-mediated linkage between two 2,4-dihydroxybenzoic acid units, are widely distributed in fungal, lichenized, and plant species. These metabolites have attracted significant research attention due to their broad-spectrum pharmacological properties, such as anti-inflammatory, antimicrobial, and free radical-scavenging capacities [4,5,6,7,8]. Our previous study discovered a structurally new depside Aspergillusidone G (Asp G), from a marine fungus *Aspergillus unguis* [9], which was also independently reported by two other groups as Unguidepside A and Aspergiside B, respectively, in 2018 [10,11]. Asp G was demonstrated to possess significant anti-neuroinflammatory capacities in lipopolysaccharide (LPS)-stimulated BV-2 cells and neuroprotective property in 1-methyl-4-phenylpyridinium (MPP^+^) injured primary neurons from mouse cerebral cortex, and the models of LPS-induced neuroinflammation and MPP^+^-induced neuronal injury have been suggested as a reliable approach to model PD-like neurodegenerative symptoms on cell level [12,13], suggesting Asp G’s potential as a therapeutic agent for Parkinson’s disease (PD).

PD, the second most prevalent neurodegenerative disorder globally, impacts 2–3% of individuals aged 65 and above [14], which is a paradoxical outcome stemming from modern society’s success in prolonging human longevity. Patients with PD experience progressive motor symptoms such as tremor, bradykinesia, and postural instability, as well as debilitating non-motor symptoms like cognitive impairment and mood disorders [15]. Current treatment strategies largely focus on symptomatic relief through dopaminergic therapies but fall short in halting disease progression. Although several interventions targeting neuroprotection are being explored, such as glutathione, nicotine, pioglitazone, granulocyte colony-stimulating factor, GM608, exercise, and surgery [16], no effective neuroprotective agents have yet been identified. Thus, novel therapeutic strategies targeting the underlying pathophysiological mechanisms, such as neuroinflammation, are urgently needed.

In PD development, neuroinflammation driven by microglial hyperactivation and the resultant overproduction of inflammatory cytokines emerges as a critical pathological mechanism [17,18,19]. Research revealed elevated peripheral and brain levels of interleukin-6 (IL-6), tumor necrosis factor (TNF), IL-1β, etc. in PD patients, providing robust clinical evidence for systemic inflammatory dysregulation in PD pathogenesis [20,21]. Down-regulation of pro-inflammatory factors alleviates PD pathology. For example, studies have shown that Pazopanib suppresses LPS-induced transcription of pro-inflammatory factors in BV2 microglia, including inducible nitric oxide synthase (iNOS), cyclooxygenase-2 (COX-2), IL-1β, and IL-6, and inhibits microglia-mediated toxicity toward murine dopaminergic MES23.5 cells [22]. In both BV2 microglial cultures and rat primary microglia, urate attenuated the release of pro-inflammatory cytokines and downregulated the expression of COX-2 and iNOS, thereby shielding dopaminergic neurons from microglia-mediated neurotoxicity [23].

Neuroinflammation, especially induced by LPS, is initiated by upstream regulators such as Toll-like receptor 4 (TLR4), nuclear factor-kappa B (NF-κB), and mitogen-activated protein kinase (MAPK), which orchestrate early inflammatory responses by activating pro-inflammatory cytokine cascades [24,25]. However, the progression of neuroinflammatory cascades relies heavily on downstream amplifiers, including matrix metalloproteinase-9 (MMP9), which exacerbates tissue damage and sustains inflammatory loops. MMP9, a member of the matrix metalloproteinase family, disrupts the blood–brain barrier integrity by degrading extracellular matrix components, facilitating leukocyte infiltration, and amplifying neuronal injury [26]. Importantly, MMP9’s role extends beyond general neuroinflammatory pathways to neurodegenerative disorders such as PD.

Elevated MMP9 levels have been observed in PD patients [27], suggesting its role as both a biomarker and a potential therapeutic target. Experimental evidence also reveals that MMP9 mediates cognitive deficits in rotenone-induced PD murine models by triggering microglial activation-driven blood–brain barrier compromise and promoting apoptotic neuronal death, establishing its pivotal role in neurotoxic pathogenesis [28]. Tolperisone HCl improves motor performance in rotenone-PD mice through MMP9 suppression and p38 MAPK/ERK1/2 downregulation [29]. These provide insight that MMP9 may play a role in the neuroinflammatory process that leads to cognitive impairment in PD, and Asp G may serve as a high-affinity ligand for MMP9.

In this context, utilizing network pharmacology, in vitro and cellular assays, we explored Asp G’s potential anti-PD mechanisms and its influence on PD-like neuroinflammatory pathology, along with its anti-inflammatory potential via MMP9 suppression. Predictions from network pharmacology showed that the potential anti-PD mechanisms of Asp G might be associated with its regulation of inflammation, and MMP9 might serve as a potential core therapeutic target. Experimentally, the capacity of Asp G to exert anti-inflammation was confirmed, and MMP9 might be Asp G’s potential target. Herein, we present our integrated bioinformatics and experimental approach to elucidate the mechanism by which Asp G alleviates neuroinflammation through MMP9 inhibition, suggesting its potential as a promising candidate for anti-neuroinflammatory drug development and potentially advancing interventive strategies for PD.

## 2. Results

### 2.1. Asp G Possesses a Great Therapeutic Potential in the Inflammatory Mechanism of PD

**Figure 1:**
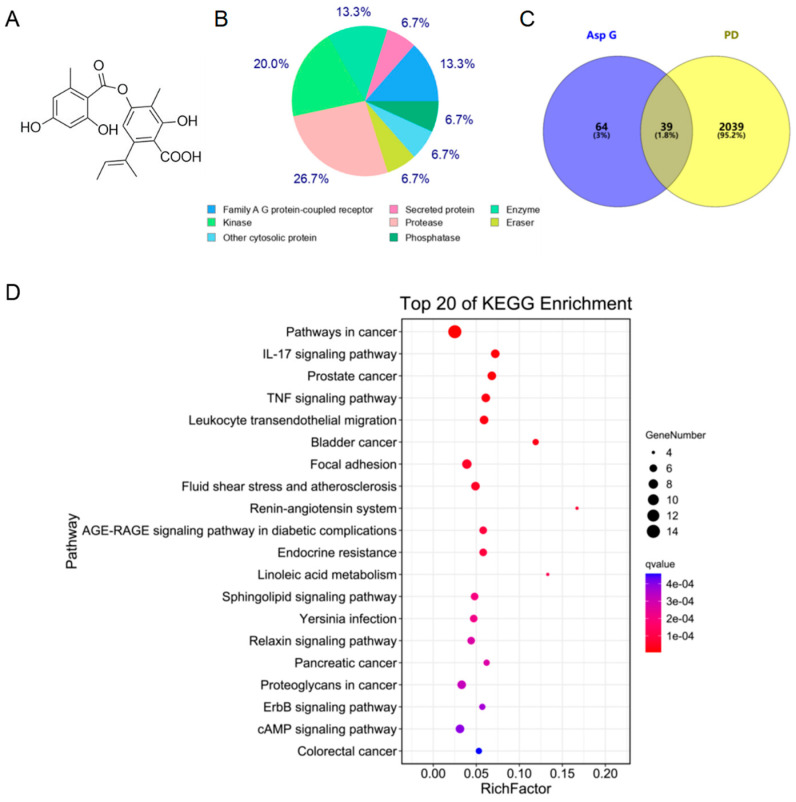
Bioinformatics prediction of Asp G against PD. (**A**) Chemical structural formula of Asp G. (**B**) Classification of Asp G-related targets from SwissTargetPrediction online website (top 50 targets). (**C**) Venn diagram visualization of intersecting molecular targets between Asp G and PD. (**D**) KEGG analysis of overlapping targets between Asp G and PD.

Our previous study showed that Asp G significantly attenuated MPP^+^-induced damage of primary cortical neurons [13], which prompted us to elucidate the molecular mechanism of Asp G against PD. Thus, we employed network pharmacology to evaluate the possible anti-PD mechanism of Asp G. Asp G’s chemical structure is shown in Figure 1A. Firstly, we predicted the targets of Asp G and downloaded PD-related targets. A total of 103 potential targets of Asp G were obtained, and most of them belong to proteases and kinases (Figure 1B). The analysis of the Venn diagram showed that more than 37% of them overlapped with PD-related targets (Figure 1C). Further, KEGG analysis showed that they are mainly involved in inflammatory pathways, such as IL-17 signaling pathway, TNF signaling pathway, leukocyte transendothelial migration, focal adhesion, etc. (Figure 1D). The above prediction indicated that inflammation might be the potential mechanism by which Asp G exerts anti-PD activity.

### 2.2. Asp G Dose-Dependently Diminishes Pro-Inflammatory Factors Expression in LPS-Induced BV2 Cells

**Figure 2:**
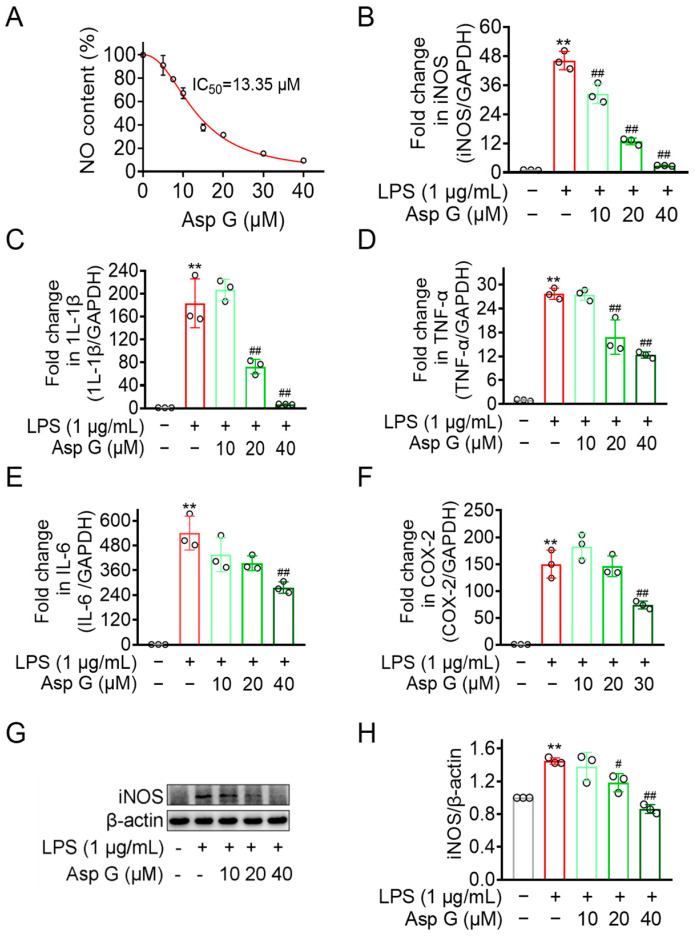
Regulatory effect of Asp G on LPS-stimulated proinflammatory responses in BV2 microglia. (**A**) The IC_50_ value of Asp G against NO production in BV2 microglia treated with LPS. The regulation of Asp G on the mRNA expression of iNOS (**B**), proinflammatory factors such as IL-1β (**C**), TNF-α (**D**), IL-6 (**E**), and COX-2 (**F**), and protein expression of iNOS (**G**,**H**) in LPS-activated BV2 cells. Quantitative data were expressed as mean ± standard deviation (*n* = 2 biological replicates for A; *n* = 3 experimental repeats for panels **B**–**H**). Open circles (○) in the bar chart refer to experimental data points. Star sign ** indicates *p* < 0.01 differentials relative to control conditions, whereas pound signs ^#^ and ^##^ mark *p* < 0.05 and *p* < 0.01 significance levels, respectively, when contrasted against LPS-treated groups.

Chronic neuroinflammation generally involves the activation of microglia, leading to the production of NO, various cytokines, and other biomarkers [19,30,31]. Our previous study has found that Asp G dose-dependently attenuated LPS-induced NO generation in BV2 microglia cells, and a low dose of Asp G (7.5 μM) promotes the attenuation of proinflammatory-related factors expression by Polaprezinc [12]. However, whether Asp G also exhibits dose-dependent alleviation for related pro-inflammatory factors has not been explored. Thus, we chose three doses of Asp G, low, medium, and high, for the following and further study. As Figure 2A shows, the IC_50_ value of Asp G against NO production was calculated as 13.35 μM in BV2 microglia exposed to LPS, with 40 μM Asp G suppressing 90.54% of the NO burst compared to the LPS group, suggesting Asp G possesses an excellent NO inhibition capability. qRT-PCR and Western blot demonstrated that Asp G suppressed both transcriptional and protein levels of iNOS, a mediator of NO synthesis (Figure 2B,G,H). Asp G additionally attenuated mRNA expression of proinflammatory cytokines IL-1β, TNF-α, and IL-6 (Figure 2C–E). COX-2, the rate-limiting enzyme for prostaglandin E2 (PGE2) production, was typically induced during inflammatory stimulation [32]. Our result showed Asp G relieved the transcriptional expression of COX-2 in LPS-induced BV2 microglia cells as well (Figure 2F). Collectively, Asp G exerted an inhibitory effect on NO generation, transcriptional expression of proinflammatory cytokines, and COX-2 in BV2 microglia cells after exposure to LPS, which confirmed the prediction that Asp G possessed anti-inflammatory activity.

### 2.3. MMP9 Was the Potential Anti-PD Target of Asp G

**Figure 3:**
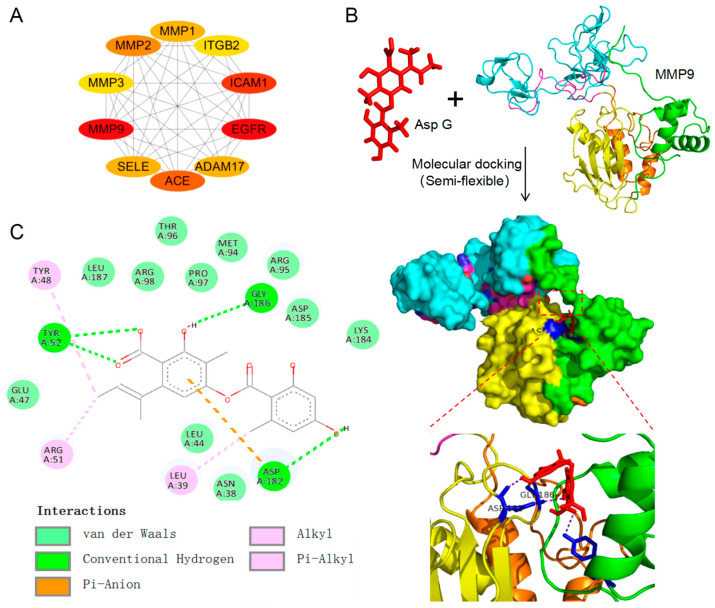
Target prediction and molecular docking analysis of potential anti-PD activity of Asp G. (**A**) Topological visualization of the PPI network hub nodes representing the ten most central overlapped targets between Asp G and PD through Cytoscape. (**B**) Molecular docking of Asp G and MMP9 by Autodock vina. MMP9 domains are shown as follows: the propeptide (green, residues: 29–109), the active site (yellow, residues: 110–215), fibronectin repeats (blue, residues: 226–273, 283–331), and Zn^2+^-binding domain (orange, residues: 390–444). (**C**) 2D diagram of interaction forces between Asp G and MMP9 by Discovery Studio.

To search for the potential target of Asp G against PD, we further performed Protein–protein interaction (PPI) network analysis of overlapping targets obtained from network pharmacology, and the result showed that MMP9 possessed the best network connectivity (Figure 3A). Moreover, the result of molecular docking revealed that Asp G formed a potentially strong interaction with the Asp 182, Gly 186, and Tyr 52 residue site of MMP9 (PDB:1L6J) (Figure 3B) by hydrogen bonds with the minimum binding energy of −8.5 kcal/mol. The analysis of protein–ligand interaction showed that there were some other interaction forces between Asp G and MMP9, including van der Waals (ASN 38, LEU 44, Glu 47, MET 94, ARG 95, THR 96, PRO 97, ARG 98, LYS 184, ASP 185, LEU 187), pi-anion (Asp 182), alkyl (Tyr 48, Arg 51), and pi-alkyl (Leu 39) (Figure 3C). The above analysis indicated there was a potential interaction between Asp G and MMP9.

### 2.4. Asp G Directly Affected the Enzymatic Activity of the Active Form of MMP9 Induced by APMA

**Figure 4:**
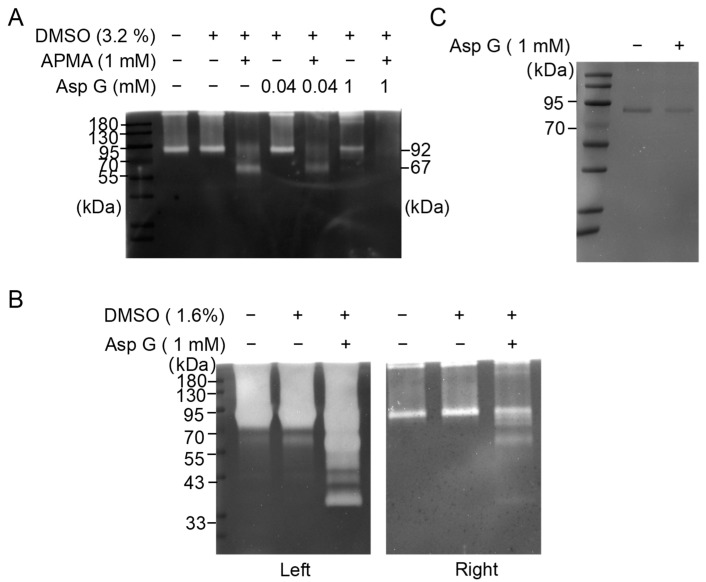
Effect of Asp G and/or APMA on in vitro MMP9 gelatinolytic activity. (**A**) Gelatinolytic activity assay of 1 ng/μL proMMP9 after co-incubation with the indicated concentrations of Asp G (0.4 mM or 1 mM) and/or 1 mM APMA at 37 °C for 24 h. Sample loading: 5 ng. (**B**) Gelatinolytic activity of proMMP9 after the co-incubation of 1.5 ng/μL proMMP9 and 1 mM Asp G at 37 °C for 24 h. Sample loading: left: 15 ng, right: 3 ng. (**C**) SDS-PAGE analysis of proMMP9 after co-incubating 50 ng/μL proMMP9 and 1 mM Asp G at 37 °C for 24 h. Sample loading: 500 ng.

To confirm whether MMP9 is a potential target of Asp G against PD, we evaluated the in vitro effect of Asp G on the gelatinolytic activity of MMP9 in its active and inactive forms based on its gelatinolytic activity by gelatin zymography, which could reflect, to some extent, the effect of Asp G on the potential proteolytic activity of MMP9 as well as the interaction between Asp G and MMP9. p-amino phenylmercuric acetate (APMA) is an organomercury agent that can be used to artificially facilitate the loss of propeptide of MMP9 through an autolytic cleavage regulated by “the cysteine switch (residues: 97–104)”, causing a conformational alteration that allows a stepwise autolytic cleavage of the propeptide, yielding an intermediate form of 83 kDa and an active species of 67 kDa [33]. In this study, it was used as an activator of recombinant human full-length proMMP9 (92 kDa). The result showed that 1 mM APMA treatment prompted the activation of 92-kDa MMP9 to its final 67-kDa form within 24 h (Figure 4A), which was consistent with Steven D. Shapiro’s research [34]. Importantly, 1 mM Asp G treatment almost eliminated the gelatinolytic activity of 67-kDa MMP9 (Figure 4A), suggesting that Asp G directly inhibited the gelatinolytic activity of the active form of MMP9 induced by APMA. Usually, the binding of drugs is proposed to stabilize target proteins, either globally or locally. However, our study found that Asp G showed a slight promotive effect on the autolytic cleavage of MMP9. As the results show, slight autocatalytic activation of MMP9 was observed in the presence of a high concentration of Asp G (1 mM) alone (Figure 4B), and the protein content of MMP9 also decreased after incubation with 1 mM Asp G (Figure 4C). Nevertheless, this phenomenon is rarely observed at a low treatment concentration (40 μM) (Figure 4A). We supposed that it was probably due to the close binding of Asp G to the catalytic site of MMP9, which pushed the cysteine residue of the prodomain slightly away from the catalytic zinc ion (Figure S3) [35]. In short, these results indicated that Asp G directly inhibited the potential proteolytic activity of the active form of MMP9.

### 2.5. Asp G Ameliorated the Production of MMP9 by Regulating Its Gene and Protein Expression in LPS-Induced BV2 Microglia

**Figure 5:**
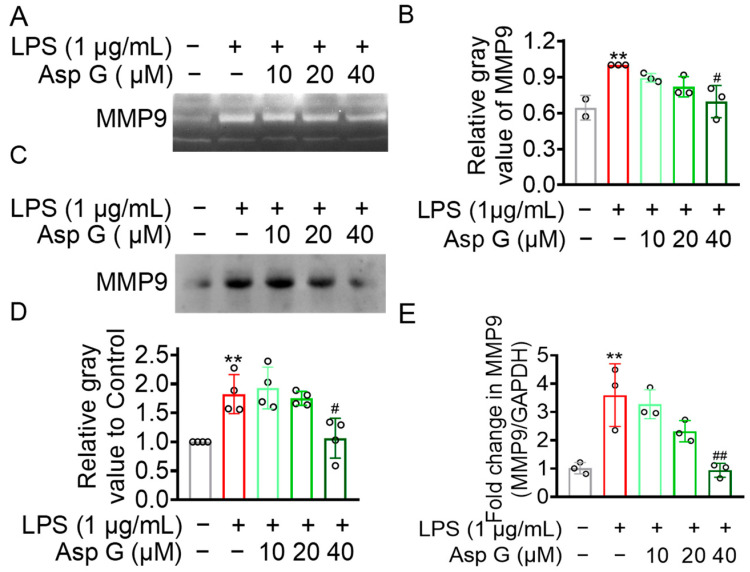
Asp G inhibited MMP9 monomers’ production in BV2 microglia after exposure to LPS. BV2 microglia cells were preincubated with the defined concentration of Asp G for 1 h prior to treatment with LPS for 24 h (**A**–**D**) or 12 h (**E**), respectively. MMP9 production was detected by gelatin zymography assay (**A**,**B**). The expression of protein (**C**,**D**) and transcription level (**E**) of MMP9 were determined by Western blot and qRT-PCR, respectively. Quantitative data were expressed as mean ± standard deviation (*n* = 3 biological replicates for (**A**,**B**,**E**); *n* = 4 experimental repeats for **C**,**D**). Open circles (○) in the bar chart refer to experimental data points. ** indicates *p* < 0.01 differentials relative to control conditions, whereas ^#^ and ^##^ indicate *p* < 0.05 and *p* < 0.01 significance levels when compared to LPS-treated groups.

It has been reported that MMP9 in vivo is found in the extracellular matrix, cerebrospinal fluid, and serum after secretion, usually presented as monomers, oligomers, complexes with other molecules, and truncated forms with low molecular weight [35]. The in vivo activity of MMP9 is generally tightly controlled at several levels, including gene transcription level, the expression and activation of inactive enzyme precursor, and the role of TIMP [36]. In in vitro experiments, we found that Asp G dramatically inhibited the gelatinolytic activity of active-form MMP9 induced by APMA, which prompted us to consider whether Asp G also exhibited a suppressive effect on the inactive form of MMP9 (MMP9 monomers), thus reducing the opportunity of the transformation of MMP9 from an inactive form to an active form. Due to the abundance of MMP9 monomers, they are also one of the most easily observed forms using gelatin zymography [35], which is consistent with the phenomenon that a larger number of MMP9 monomers were detected in the liquid supernatant of LPS-induced BV2 microglia. Our study found that LPS treatment strikingly increased the production of extracellular MMP9 monomer, and this rise was distinctly suppressed under the treatment of Asp G (Figure 5A,B and Figure S1). Importantly, Asp G treatment significantly mitigated the intracellular increased mRNA (Figure 5C,D) and extracellular protein (Figure 5E and Figure S2) level of MMP9 in LPS-induced BV2 microglia, suggesting that Asp G decreased the production of MMP9 monomers by inhibiting its gene transcription and protein expression level in LPS-induced BV2 cells.

### 2.6. Asp G Attenuated the Production of Inflammation-Related Factors by Regulating MMP9 in LPS-Induced BV2 Microglia

**Figure 6:**
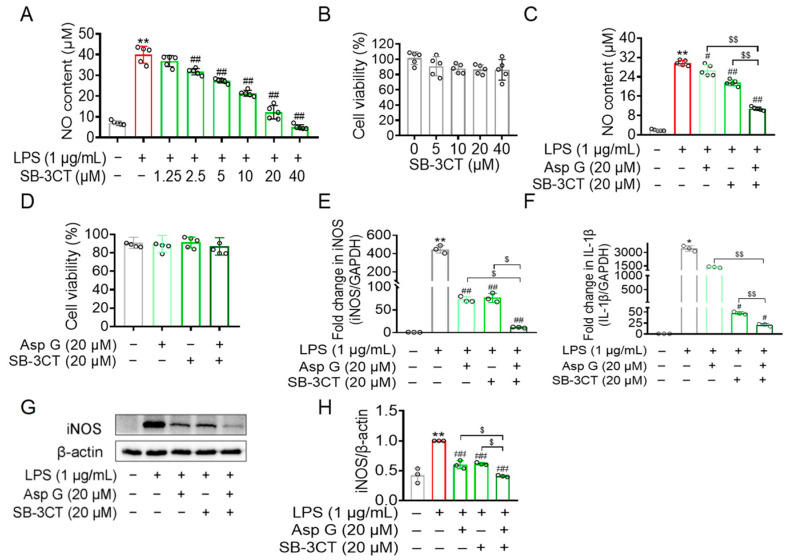
The role of MMP9 in Asp G inhibiting LPS-induced inflammation-related factors in BV2 cells. BV2 microglia cells were pretreated with SB-3CT for 30 min and/or Asp G for 1 h, followed by stimulation with LPS for another 12 h (**E**,**F**) and 24 h (**A**,**C**,**G**,**H**), respectively. (**A**) The NO content of BV2 microglia cells pretreated with the different concentrations of SB-3CT, followed by stimulation with LPS, was detected by Griess reagent. (**B**) Cell viability of BV2 microglia after exposure to a series of SB-3CT for 24 h was tested using CCK8. (**C**) The NO content of BV2 microglia cells pretreated with SB-3CT and/or Asp G, followed by treatment with LPS, was determined using Griess reagent. (**D**) Cell viability of BV2 microglia after exposure to SB-3CT and/or Asp G for 24 h was tested using CCK8. qRT-PCR was used to determine the transcriptional expression of iNOS (**E**) and 1L-1β (**F**) in BV2 microglia after exposure to Asp G and/or SB-3CT and LPS. Western blot was performed to detect the protein expression of iNOS (**G**,**H**) in BV2 microglia after the stimulation of Asp G and/or SB-3CT and LPS. Quantitative data were expressed as mean ± standard deviation (*n* = 3 biological replicates for (**E**–**H**); *n* = 5 experimental repeats for **A**–**D**). Open circles (○) in the bar chart refer to experimental data points. * and ** indicate *p* < 0.05 and *p* < 0.01 differentials, respectively, relative to control condition; ^#^ and ^##^ indicate *p* < 0.05 and *p* < 0.01 significance levels, respectively, when compared to LPS-treated group; ^$^ and ^$$^ represent *p* < 0.05 and *p* < 0.01 significance levels, respectively, when compared to LPS_Asp G_SB-3CT group.

To further explore whether the aforementioned anti-inflammatory ability of Asp G depends on its inhibitory effect on MMP9, a competitive MMP2 and MMP9 inhibitor (SB-3CT) was used here. SB-3CT has high selectivity for gelatinases and binds to the active sites of MMP2 and MMP9. The thiirane moiety of SB-3CT coordinates with the active site zinc ion of gelatinases, and this coordination activates the thiirane ring of SB-3CT for opening by the nucleophilic attack of the active site glutamate of gelatinases, causing the generation of an inhibitory species composed of the inhibitor and the enzyme by a tight binding [37,38]. Our result showed that the treatment of SB-3CT (1.25–40 μM) dose-dependently reduced LPS-induced NO production in BV2 cells (Figure 6A) without exhibiting cytotoxicity (Figure 6B). The co-treatment of 20 μM Asp G and 20 μM SB-3CT exhibited a more effective reduction in LPS-induced NO generation than either Asp G or SB-3CT alone, with the inhibition rate on NO increasing from 27.57% to 63.50% compared to the LPS group (Figure 6C), without exhibiting cytotoxicity (Figure 6D). The co-treatment significantly suppressed both the mRNA level of iNOS (Figure 6E) and 1L-1β (Figure 6F), along with the protein level of iNOS (Figure 6G,H), revealing that Asp G might suppress LPS-triggered microglia activation via inhibiting MMP9 in BV2 microglia.

## 3. Discussion

### 3.1. Anti-Neuroinflammatory Mechanism of Asp G: An Implication for PD Intervention

The global prevalence of PD imposes a substantial socioeconomic burden. Current clinical therapies merely alleviate symptoms with severe side effects, whereas natural products emerge as promising candidates due to their multi-target capacity and low toxicity [39]. Here, we identified Asp G, a marine fungus-derived depside, as a potent anti-neuroinflammatory agent, providing an implication for PD therapeutics.

Network pharmacology revealed that some targets of Asp G overlapped with those in PD, and these shared targets are mainly involved in the IL-17 signaling pathway, TNF signaling pathway, leukocyte transendothelial migration, and focal adhesion. Previous mechanistic studies have confirmed the relevance of these pathways to PD pathology. For instance, Sommer et al. demonstrate that T cells secreting IL-17 in sporadic PD patients induce neuronal death in midbrain neurons derived from patient-specific induced pluripotent stem cells [40]. Additionally, TNF-α [41], leukocyte transendothelial migration [42], and focal adhesion [43] signaling pathways have also been shown to be associated with PD pathology.

As we know, these pathways mentioned above are primarily involved in inflammatory pathology. Specifically, the IL-17/TNF pathways directly drive the release of proinflammatory factors and amplify inflammatory signaling [44]; leukocyte transendothelial migration serves as an essential process facilitating the infiltration of inflammatory cells into tissues [45]; focal adhesion modulates the adhesion and migration of inflammatory cells through integrin-mediated mechanisms [46], indicating that Asp G may be associated with inflammatory cascades in PD. Experimentally, Asp G dose-dependently suppressed LPS-induced NO release and significantly downregulated the expression of iNOS, COX-2, and proinflammatory factors (IL-6, TNF-α, IL-1β). Research indicates that levels of pro-inflammatory cytokines (TNF, IL-1β, IL-6, etc.) in the brain are elevated and correlate with disease severity and disability in the serum of patients with PD [20,47,48], and their down-regulated expression can significantly inhibit the development of PD. For example, iNOS-knockout mice exhibited enhanced resistance to MPTP-induced neurotoxicity relative to their WT littermates [49], and adalimumab-mediated TNF-α blockade suppressed gastrointestinal and central nervous system inflammation, attenuated nigrostriatal degeneration, and rescued motor deficits in LRRK2 mutant (G2019S) mice with colitis [41]. Thus, the anti-neuroinflammatory effect of Asp G positions this marine-derived compound as a promising therapeutic candidate for combating neuroinflammatory processes implicated in PD pathogenesis.

Our cell experiments adopted a prophylactic regimen, with Asp G administered 1 h prior to LPS exposure, suggesting that Asp G may exert anti-neuroinflammatory effects by potentially blocking LPS-associated receptors such as TLR4. Studies indicate TLR4 plays a pivotal role in LPS-induced neuroinflammation by initiating downstream proinflammatory cascades [50]. However, PD-related neuropathology predominantly stems from sustained neuroinflammatory amplification [51], where downstream effectors like MMP9 critically exacerbate tissue damage and perpetuate inflammatory cycles [28,52]. Targeted inhibition of such amplifiers could therefore provide crucial points for disrupting PD progression. Considering that TLR4 may also serve as an upstream target mediating the anti-inflammatory effects of Asp G, our future studies will employ a TLR4 inhibitor like TAK242 to quantify Asp G’s effects on LPS-induced activation of TLR4, NF-κB, and MAPK, evaluating its dual regulatory potential in neuroinflammatory control. This multilayered approach aligns with emerging strategies for optimizing PD intervention.

### 3.2. MMP9 Is a Potential Therapeutic Target of Asp G

Predictions from network pharmacology support the relationship between Asp G and the neuroinflammatory cascade. The analysis of PPI and autodock docking indicated that the target of MMP9 with the best network connectivity possessed a potentially strong interaction with Asp G by hydrogen bonds and other interaction forces, suggesting that MMP9 might be the potential target of Asp G against PD. This aligns with Zhao et al., where genistein nanoparticles showed high-affinity binding to MMP9’s catalytic domain, suppressing extracellular matrix degradation through MMP9 inhibition [53]. In PD pathology, MMP9 is implicated in blood–brain barrier disruption and dopaminergic neurodegeneration. Research indicates that MMP2/9-dependent blood–brain barrier disruption mediates microglia-driven dopaminergic neurodegeneration in the rotenone-induced PD murine model [54]. In addition, MMP9-dependent cleavage of β-dystroglycan impairs glymphatic clearance via disruption of AQP4 polarization in MPTP-induced PD and A53T mice [55]. Experimentally, 1 mM Asp G nearly abolished APMA-induced gelatinolytic activity of 67-kDa MMP9, likely due to either autolysis into inactive fragments or structural/conformational disruption by Asp G, rather than blocking proMMP9 activation. Notably, unlike TIMP-1 inhibitors requiring the MMP9 carboxyterminal domain for binding [56,57], Asp G effectively inhibited truncated 67-kDa MMP9 devoid of this domain, highlighting its unique therapeutic advantage. Collectively, the robust binding affinity between Asp G and MMP9, coupled with its direct suppression of MMP9’s gelatinolytic function in vitro, positions MMP9 as a potential target mediating Asp G’s anti-Parkinsonian effects.

Additionally, our study found that, compared to the LPS group, Asp G distinctly suppressed the increase of extracellular monomer, intracellular mRNA, and extracellular protein level of MMP9 in LPS-induced BV2 microglia. These findings suggested Asp G also exhibited a suppressive effect on the inactive form of MMP9, thereby reducing the opportunity for its conversion from the inactive to the active form. Consistent with Lee et al., β-LAP suppresses LPS-induced MMP9 upregulation transcriptionally and translationally in both BV2 and primary microglial models, reinforcing its role in neuroinflammatory modulation [58]. Furthermore, the combination of Asp G and SB-3CT demonstrated a significantly greater reduction in LPS-induced NO production, along with decreased mRNA levels of iNOS and IL-1β, and suppressed iNOS protein expression, compared to either agent administered alone. SB-3CT, a selective MMP2/9 inhibitor, has been shown to mitigate rotenone-induced cognitive deficits in mice by targeting microglia-mediated blood–brain barrier disruption and neuronal apoptosis via MMP2/9, highlighting its mechanistic relevance to PD pathogenesis [28]. Taken together, Asp G may further attenuate neuroinflammation implicated in PD intervention by indirectly suppressing MMP9 expression.

While these preliminary findings are encouraging, further studies are needed to fully address the inherent limitations of this exploratory work. For instance, in vitro validation of molecular interactions between Asp G and MMP9 by surface plasmon resonance (SPR), fluorescence resonance energy transfer (FRET), isothermal titration calorimetry (ITC), etc., requires further validation. In addition, the upstream regulatory mechanisms of Asp G-mediated MMP9 inhibition and further in vivo animal experiments are needed in future studies to comprehensively evaluate the anti-PD potential and mechanism of Asp G.

## 4. Materials and Methods

### 4.1. Reagents

Asp G (purity: HPLC ≥ 97%) was isolated and purified from the marine fungus *Aspergillus unguis* DLEP2008001 following the method previously reported [9]. Asp G dried powder was stored as dry powder in amber glass bottles at −80 °C and dissolved in anhydrous DMSO (196055, MP Biomedicals, Irvine, CA, USA) before use.

### 4.2. Cell Line and Culture Conditions

BV2 murine microglia, originally generated in 1990 by E. Blasi through retroviral transfection of mouse microglia with *v-raf/v-myc* oncogenes, were obtained from the China Center for Type Culture Collection (GDC0311, Wuhan, Hubei, China). These cells retain native microglial morphological, phenotypic, and functional properties [59]. Cultures were maintained in Dulbecco’s Modified Eagle’s Medium (DMEM, C11995500BT, Thermo Fisher Scientific, Waltham, MA, USA) supplemented with 10% fetal bovine serum (FBS, Z7186FBS, Zeta Life, Menlo Park, CA, USA) and 1% (*v*/*v*) penicillin-streptomycin antibiotic mixture (15140122, Life Technologies, Carlsbad, CA, USA), under standard incubation conditions (37 °C, 5% CO_2_).

### 4.3. Network Pharmacology Prediction

A network pharmacology approach was employed to investigate Asp G’s anti-PD therapeutic potential and mechanisms [60]. Human targets of Asp G were predicted using the SwissTargetPrediction database (http://www.swisstargetprediction.ch/) by submitting Asp G’s SMILES identifier accessed on 4 August 2022, while PD-associated targets were retrieved from the Disgenet online website (https://www.disgenet.org/) with “Parkinson’s disease” as the query accessed on 4 August 2022. Common targets shared between Asp G and PD were identified by Venny 2.1 (https://bioinfogp.cnb.csic.es/tools/venny/) accessed on 4 August 2022. Functional enrichment analysis of these overlapping targets, including Gene Ontology (GO) and Kyoto Encyclopedia of Genes and Genomes (KEGG) pathways, was performed using the R package clusterProfiler [61]. PPI networks were constructed from the STRING database (https://cn.string-db.org/) accessed on 6 August 2022, with core targets selected based on topological network metrics and visualized in Cytoscape_v3.9.1.

### 4.4. Molecular Docking

The potential interactions of Asp G and MMP9 were analyzed through semiflexible molecular docking with AutoDock Vina 1.2.3 [62]. The MMP9 structural file (1L6J.pdb) was retrieved from the PDB database (https://www.rcsb.org/) accessed on 18 October 2023, and the Asp G structure was generated using ChemDraw 20.0 and Pymol 3.0.3. Redundant ligands, water molecules, and non-essential components were eliminated from the MMP9 system, followed by the incorporation of hydrogen atoms to generate its .qdbqt coordinate files. For Asp G, active key numbers were assigned to produce the corresponding .pdbqt files. The spatial search range for ligand binding was defined by configuring the “grid box” parameters (center coordinates: x = 36.885, y = 38.845, z = 34.621; dimensions: x = 89.95, y = 89.95, z = 89.95). AutoDock Vina was then executed using the preconfigured settings. The resultant docking output in .pdbqt format was converted to a .pdb file. Subsequently, the ligand and receptor structures were merged and subjected to visual inspection with Pymol 3.0.3. Two-dimensional representations of ligand-protein interactions were generated using Discovery Studio 2021.

### 4.5. Griess Assay

NO levels in cell culture supernatants were quantified using a commercial detection kit (S0021, Beyotime Biotech, Shanghai, China) following standardized protocols [63]. BV2 microglia cells were seeded in 96-well plates at 2 × 10^4^ cells/well and cultured for 24 h. The experimental sequence for administering SB-3CT, Asp G, and LPS is structured as follows: cells were initially primed with SB-3CT (30 min), subsequently exposed to Asp G (1 h), and ultimately challenged with LPS (24 h), with all treatments performed consecutively in the same medium. A total of 30 μL of supernatant was combined with equal volumes of Griess reagents I and II. Absorbance at 540 nm was measured using a microplate reader (Epoch2, Biotek, Winooski, VT, USA), with NO concentrations determined through sodium nitrite standard curves.

### 4.6. CCK-8 Assay

Cell viability was assessed using the CCK-8 assay following standardized protocols [64]. BV2 microglia were seeded in 96-well plates (10^4^ cells/well) for 24 h. Cells were then treated with specified concentrations of Asp G and/or SB-3CT in fresh DMEM for an additional 24 h. Following incubation, 10 μL of CCK-8 reagent (CCK-8, K009, Zeta Life, Menlo Park, CA, USA) mixed with 90 μL DMEM was added to each well and incubated at 37 °C for 1 h. Optical density at 450 nm was quantified using a microplate reader.

### 4.7. Quantitative Real-Time PCR (qRT-PCR)

BV2 microglia were cultured in 6-well plates (6 × 10^5^ cells/well) for 24 h. The sequential treatment order of the three agents, including SB-3CT, Asp G, and LPS, is as follows: cells were first pretreated with SB-3CT for 30 min, then followed by Asp G for 1 h, and finally stimulated with LPS for 12 h, with no medium replacement between any steps. Total RNA extraction was performed with TRIzol^®^ reagent (AG21101, Accurate Biology, Changsha, Hunan, China) following established protocols [65]. RNA integrity and quantification were verified using a DeNovix DS-11 spectrophotometer (DS-11, DeNovix, Wilmington, DE, USA). Qualified RNA samples underwent reverse transcription to cDNA using HiScript III RT SuperMix for qPCR (+gDNA wiper) (R323, Vazyme, Nanjing, Jiangsu, China) following the manufacturer’s instructions. Quantitative real-time PCR analysis was conducted with ChamQ Universal SYBR qPCR Master Mix (Q711, Vazyme, Nanjing, Jiangsu, China) on a Real-Time PCR Detection System (CFX96 Touch, Bio-Rad, Hercules, CA, USA). Gene expression levels were normalized to GAPDH and calculated via the 2^−ΔΔCt^ method. Primer sequences employed in this analysis are listed below (5′→3′): GAPDH: F-AGGTCGGTGTGAACGGATTTG, R-TGTAGACCATGTAGTTGAGGTCA; COX-2: F-TTCAACACACTCTATCACTGGC; R-AGAAGCGTTTGCGGTACTCAT; IL-6: F-TAGTCCTTCCTACCCCAATTTCC, R-TTGGTCCTTAGCCACTCCTTC; IL-1β: F-GCAACTGTTCCTGAACTCAACT, R-ATCTTTTGGGGTCCGTCAACT; TNF-α: F-CCCTCACACTCAGATCATCTTCT, R-GCTACGACGTGGGCTACAG; iNOS: F-GTTCTCAGCCCAACAATACAAGA, R-GTGGACGGGTCGATGTCAC; MMP9: F-CTGGACAGCCAGACACTAAAG, R-CTCGCGGCAAGTCTTCAGAG.

### 4.8. Western Blot

BV2 microglia were cultured in 6 cm dishes (1.3125 × 10^6^ cells/dish) for 24 h. The three agents, including SB-3CT, Asp G, and LPS, were administered sequentially as follows: cells underwent an initial pre-incubation with SB-3CT for 30 min, followed by exposure to Asp G for 1 h, and concluding with LPS stimulation for 24 h. All treatments were performed in the same culture medium without interim replacement. For MMP9 analysis, intracellular proteins were lysed using ice-cold EDTA-free RIPA buffer (50 mM Tris-HCl (pH 7.4), 1% (*v*/*v*) Triton X-100, 1% (*w*/*v*) sodium deoxycholate, 0.1% (*w*/*v*) SDS) supplemented with 1 mM PMSF (ST506, Beyotime Biotechnology, Shanghai, China) to prevent metal chelator interference, while extracellular proteins from supernatant were then concentrated by lyophilization and dissolved in ice-cold EDTA-free RIPA buffer. Other protein targets required cell lysis in ice-cold RIPA buffer (P0013B, Beyotime Biotechnology, Shanghai, China) containing 1 mM of PMSF. Protein quantification was performed using a BCA protein assay kit (P0010S, Beyotime Biotechnology, Shanghai, China) as per the manufacturer’s guidelines. Western blot analysis was performed following the previous methodology [66]. In brief, denatured protein aliquots of equal quantity were electrophoretically resolved on 10% SDS-polyacrylamide gels and subsequently transferred onto Amersham^TM^ nitrocellulose membranes (10600001, Cytiva, Meriden, CT, USA). Membranes underwent blocking with 5% (*w*/*v*) skimmed milk for 2 h at room temperature. Sequential incubations were then conducted with species-specific primary antibodies at 4 °C for 12–16 h followed by corresponding HRP-linked secondary antibodies (2 h, room temperature). Protein-antibody complexes were visualized using a hypersensitive ECL chemiluminescent reagent (F03, Willget Biotechnology, Hangzhou, Zhejiang, China). Band intensity quantification was conducted using ImageJ 1.53q (National Institutes of Health, Bethesda, MD, USA). Antibody specifications as follows: Mouse anti-β-actin lgG (sc-47778, Santa Cruz Biotechnology, Dallas, TX, USA); Mouse anti-Nos2 lgG (sc-7271, Santa Cruz Biotechnology, Dallas, TX, USA); Rabbit anti-MMP9 pAb (WL03096, Wanleibio, Shenyang, Liaoning, China); Horseradish peroxidase (HRP)-coupled goat anti-rabbit lgG (A21020, Abbkine, Atlanta, GA, USA); Horseradish peroxidase (HRP)-coupled horse anti-mouse lgG (7076, Cell Signaling Technology, Danvers, MA, USA).

### 4.9. Gelatin Zymography

Gelatin zymography was carried out to detect the gelatinolytic activity of MMP9 [67,68]. Briefly, BV2 microglia were seeded in a 6 cm Petri dish at a density of 1.3125 × 10^6^/dish for 24 h, and then pretreated with Asp G for 1 h, followed by LPS for 24 h. Next, cells were cracked in ice-cold EDTA-free RIPA buffer containing 1 mM PMSF to obtain intracellular total protein. The BCA method was used to detect and calculate the protein concentration. The supernatant was collected and freeze-dried to get extracellular total protein, and then the protein was concentrated by lyophilization and redissolved with an equal volume of EDTA-free RIPA buffer containing 1 mM PMSF. SDS-PAGE was performed to separate these protein samples in 1 × non-reducing loading buffer (250 mM Tris-HCl (*w*/*v*), 10% SDS (*w*/*v*), 0.5% (*w*/*v*) bromophenol blue, 50% (*v*/*v*) glycerin) that had not been heat-denatured using standard 10% polyacrylamide gels containing 0.1% (*w*/*v*) gelatin. After electrophoresis, the gels were eluted twice for 40 min with elution buffer (2.5% (*v*/*v*) Triton X-100, 50 mM Tris, 5 mM CaCl_2_, 1 μM ZnCl_2_, pH = 7.6) to remove SDS. Subsequently, the gels were washed twice with washing buffer (50 mM Tris, 5 mM CaCl_2_, 1 μM ZnCl_2_, pH = 7.6) for 20 min, and then incubated with incubation buffer (50 mM Tris, 150 mM NaCl, 10 mM CaCl2, 1 μM ZnCl_2_, 0.02% (*w*/*v*) Brij-35, pH = 7.6) for gelatin degradation for 2 days at 37 ˚C. Finally, the gels were stained with the Coomassie Brilliant Blue R-250, and the densities of the bands were analyzed with Image J 1.53q.

### 4.10. In Vitro Activity Assay of MMP9

The in vitro interaction between Asp G and recombinant human full-length proMMP9 (CI71, Novoprotein, Suzhou, Jiangsu, China) was determined as follows. Briefly, 1 ng/μL proMMP9 was co-incubated with the indicated concentration of Asp G (0.4 mM or 1 mM) and/or 1 mM APMA (KP1411, KKL Med, Ashland, VA, USA) for 24 h. Then the incubation solution was examined by SDS-PAGE, elution, wash, incubation, and staining as described in the gelatin zymography assay.

### 4.11. Statistics Analysis

All experimental data were expressed as mean ± standard deviation (SD) and analyzed using GraphPad Prism 8.0.1. Normality was verified by the Shapiro–Wilk test (α = 0.05), and homogeneity of variance was confirmed via the Brown–Forsythe test. Statistical comparisons among multiple groups were performed by one-way analysis of variance (ANOVA) followed by Tukey’s post hoc test for pairwise comparisons. A threshold of *p* < 0.05 was predefined to determine statistical significance.

## 5. Conclusions

This study reveals that Asp G, a depside extracted from the marine fungus *Aspergillus unguis* DLEP2008001, attenuates neuroinflammation via inhibiting MMP9. The analysis of network pharmacology shows that inflammation is probably a mechanism for Asp G against PD, and MMP9 may be a potential anti-PD target of Asp G. The findings confirm that Asp G dramatically reduces the overproduction of LPS-stimulated proinflammatory-related mediators and factors. Notably, 40 μM Asp G exhibited a 90.54% inhibition of the NO burst relative to the LPS-treated group. The anti-inflammatory effect may be attributed to the inhibition of MMP9 by Asp G, as Asp G inhibits MMP9 production and the potential proteolytic activity of 67 kDa-MMP9. More importantly, targeted inhibition of MMP9 using 20 μM SB-3CT augmented the anti-inflammatory efficacy of 20 μM Asp G, as evidenced by a marked rise in NO suppression from 27.57% to 63.50% compared to the LPS-treated group. Further investigations will explore the upstream mechanisms underlying Asp G’s inhibition of MMP9 and evaluate its efficacy in in vivo models of PD. In short, our research demonstrates that Asp G alleviates neuroinflammation through MMP9 inhibition, suggesting its potential as a lead compound for anti-neuroinflammatory drug development and also provides an implication for interventive strategies for PD.

## Data Availability

The data presented in this study are available upon request from the corresponding authors.

## Supplementary Materials

The following supporting information can be downloaded at: https://www.mdpi.com/article/10.3390/md23050181/s1, Figure S1: Asp G mainly inhibited the gelatinolytic activity of extracellular MMP9 in LPS-induced BV2 microglia; Figure S2: Asp G mainly inhibited the protein expression of extracellular MMP9 in LPS-exposed BV2 microglia; Figure S3: The interaction of Asp G with the three residue sites (Tyr 52, Gly 186, and Asp 182) through hydrogen bonds.
