# Supplementary material for: Aspergillusidone G Exerts Anti-Neuroinflammatory Effects via Inhibiting MMP9 Through Integrated Bioinformatics and Experimental Analysis: Implications for Parkinson’s Disease Intervention
Source: Mar Drugs. 2025 Apr 23;23(5):181. doi: 10.3390/md23050181 (PMC12113303; doi:10.3390/md23050181)
Supplement: Supplementary file 1 [file marinedrugs-23-00181-s001.zip › marinedrugs-3568405-supplementary.pdf]

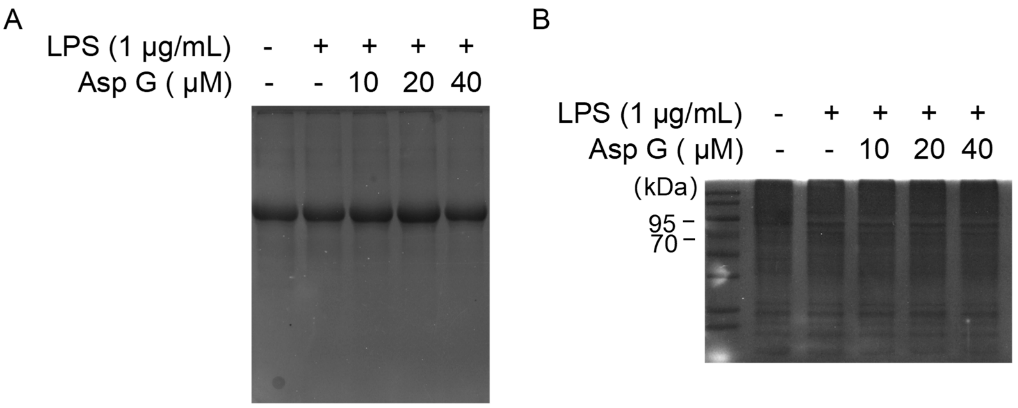

Figure S1. Asp G mainly inhibited the gelatinolytic activity of extracellular MMP9 in LPS-induced BV2 microglia. SDS-PAGE analysis of extracellular total protein (A) and gelatinolytic activity of intracellular MMP9 (B) in BV2 microglia pretreated with the indicated concentration of Asp G for 1 h, followed by exposure to 1 µg/mL LPS for another 24 h.

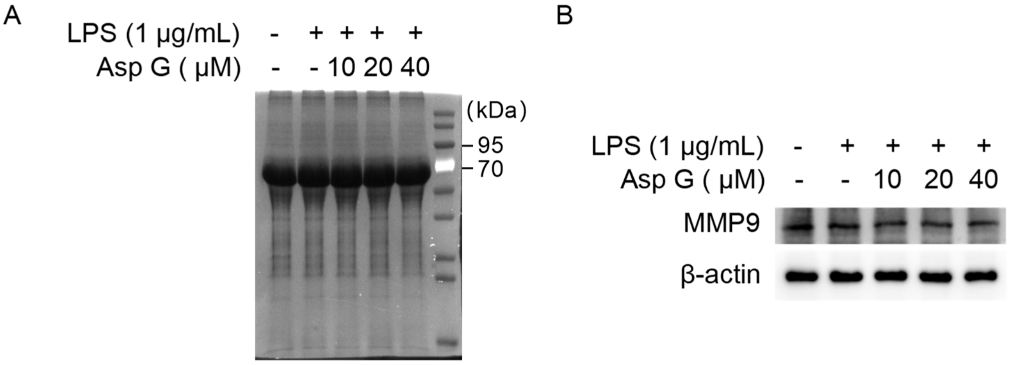

Figure S2. Asp G mainly inhibited the protein expression of extracellular MMP9 in LPS-exposed BV2 microglia. SDS-PAGE analysis of extracellular total protein (A) and Western blot analysis of intracellular MMP9 protein level (B) in BV2 microglia exposed to the different concentrations of Asp G for 1 h prior to the treatment of 1 µg/mL LPS for another 24 h.

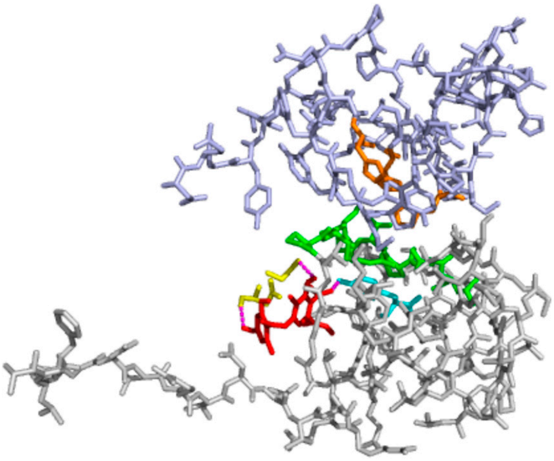

Figure S3. The interaction of Asp G with the three residue sites (Tyr 52, Gly 186, and Asp 182) of MMP9 through hydrogen bonds. Molecules indicated by different colors as follows: red: Asp G, green: "cysteine switch", blue: Tyr 52 residue in propeptide domain, yellow: Gly 186 and Asp 182 residues in active site domain, orange: Zn^{2+}-binding site (described in UniprotKB database, including His 401, His 405 and His 411), light blue: Zn^{2+}-binding domain, gray: the remaining residues of MMP9 prodomain.
